# Amyloid-β plaque formation and reactive gliosis are required for induction of cognitive deficits in *App* knock-in mouse models of Alzheimer’s disease

**DOI:** 10.1186/s12868-019-0496-6

**Published:** 2019-03-20

**Authors:** Yasufumi Sakakibara, Michiko Sekiya, Takashi Saito, Takaomi C. Saido, Koichi M. Iijima

**Affiliations:** 1 Department of Alzheimer’s Disease Research, Center for Development of Advanced Medicine for Dementia, National Center for Geriatrics and Gerontology, Obu, Aichi 474-8511 Japan; 2 Laboratory for Proteolytic Neuroscience, RIKEN Center for Brain Science, Wako, Saitama 351-0198 Japan; 3 Department of Experimental Gerontology, Graduate School of Pharmaceutical Sciences, Nagoya City University, Nagoya, 467-8603 Japan

**Keywords:** Alzheimer’s disease, Amyloid precursor protein, Knock-in mouse model, Cognitive deficits, Neuroinflammation

## Abstract

**Background:**

Knock-in (KI) mouse models of Alzheimer’s disease (AD) that endogenously overproduce Aβ without non-physiological overexpression of amyloid precursor protein (APP) provide important insights into the pathogenic mechanisms of AD. Previously, we reported that *App*^*NL*-*G*-*F*^ mice, which harbor three familial AD mutations (Swedish, Beyreuther/Iberian, and Arctic) exhibited emotional alterations before the onset of definitive cognitive deficits. To determine whether these mice exhibit deficits in learning and memory at more advanced ages, we compared the Morris water maze performance of *App*^*NL*-*G*-*F*^ and *App*^*NL*^ mice, which harbor only the Swedish mutation, with that of wild-type (WT) C57BL/6J mice at the age of 24 months. To correlate cognitive deficits and neuroinflammation, we also examined Aβ plaque formation and reactive gliosis in these mice.

**Results:**

In the Morris water maze, a spatial task, 24-month-old *App*^*NL*-*G*-*F/NL*-*G*-*F*^ mice exhibited significantly poorer spatial learning than WT mice during the hidden training sessions, but similarly to WT mice during the visible training sessions. Not surprisingly, *App*^*NL*-*G*-*F/NL*-*G*-*F*^ mice also exhibited spatial memory deficits both 1 and 7 days after the last training session. By contrast, 24-month-old *App*^*NL/NL*^ mice had intact spatial learning and memory relative to WT mice. Immunohistochemical analyses revealed that 24-month-old *App*^*NL*-*G*-*F/NL*-*G*-*F*^ mice developed massive Aβ plaques and reactive gliosis (microgliosis and astrocytosis) throughout the brain, including the cortex and hippocampus. By contrast, we observed no detectable brain pathology in *App*^*NL/NL*^ mice despite overproduction of human Aβ40 and Aβ42 in their brains.

**Conclusions:**

Aβ plaque formation, followed by sustained neuroinflammation, is necessary for the induction of definitive cognitive deficits in *App*-KI mouse models of AD. Our data also indicate that introduction of the Swedish mutation alone in endogenous APP is not sufficient to produce either AD-related brain pathology or cognitive deficits in mice.

**Electronic supplementary material:**

The online version of this article (10.1186/s12868-019-0496-6) contains supplementary material, which is available to authorized users.

## Background

Authentic animal models for Alzheimer’s disease (AD) research are of vital importance for investigating the molecular mechanisms and testing potential therapeutic approaches to AD [1, 2]. Several transgenic mouse lines overexpressing amyloid precursor protein (APP) that recapitulate amyloid-β (Aβ) deposition and the accompanying behavioral deficits have been instrumental to AD research [3–6]. However, these mice may also exhibit phenotypes because they overproduce various APP fragments in addition to Aβ [7–9].

To overcome this problem, alternative mouse models have been generated via knock-in (KI) of a humanized Aβ sequences harboring familial AD mutations (Swedish (NL), Beyreuther/Iberian (F), and Arctic (G)) in order to model Aβ amyloidosis without non-physiological overexpression of APP [10]. In *App*^*NL*-*G*-*F*^ mice, which harbor all three mutations, Aβ amyloidosis is aggressive, and neuroinflammation is observed in subcortical structures as well as cortical regions [10–12]. By contrast, *App*^*NL*^ mice that carry only the Swedish mutation produce significantly higher levels of Aβ40 and Aβ42 without overt AD-related brain pathology such as extracellular Aβ plaques or neuroinflammation [10, 11, 13]. None of these *App*-KI mice exhibit tau pathology or severe neuronal loss, suggesting that they are suitable models for preclinical AD [9].

Previously, we reported that *App*^*NL*-*G*-*F*^ mice exhibit anxiolytic-like phenotypes in the elevated plus maze task at 6 months of age, as well as a subtle decline in spatial learning ability during the acquisition session of the Barnes maze task at 8 months [14]. These results suggest that *App*^*NL*-*G*-*F*^ mice develop emotional alterations prior to the emergence of the definitive cognitive deficits. By contrast, *App*^*NL*^ mice do not undergo overt cognitive decline prior to 8 months of age [14], although it remains to be seen whether these *App*-KI mice exhibit learning and memory deficits at more advanced ages.

In this study, we assessed the performance of *App*^*NL*-*G*-*F*^, *App*^*NL*^, and wild-type (WT) C57BL/6J mice in a spatial task at the age of 24 months. To correlate cognitive deficits and neuroinflammation, we also examined Aβ plaque formation and reactive gliosis in these mice. Our results demonstrate that Aβ deposits, followed by sustained neuroinflammation, is required for induction of definitive cognitive deficits in *App*-KI mouse models of AD.

## Results

### *App*^*NL*-*G*-*F/NL*-*G*-*F*^ mice exhibit spatial learning deficits and reduced memory function in the Morris water maze task

**Fig. 1:**
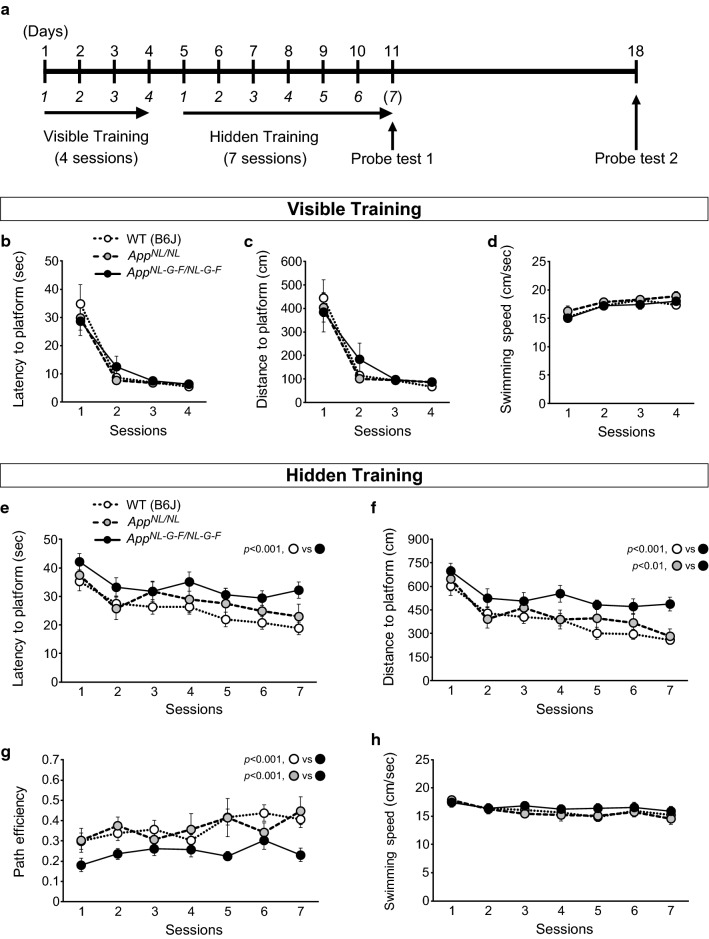
Learning performance in *App*^*NL*-*G*-*F/NL*-*G*-*F*^ and *App*^*NL/NL*^ mice during training sessions in the Morris water maze task. **a** Schematic timeline of the Morris water maze (MWM) task. Mice received visible training for 4 days (4 trials per day) to assess motor and visual capabilities, followed by hidden training for 7 days (4 trials per day) to assess spatial learning ability. Two probe tests were conducted at 1 day after the sixth session (Probe test 1) and at 7 days after the seventh session of the hidden training (Probe test 2) to assess spatial memory performance. **b**–**d** In the visible training sessions, *App*^*NL*-*G*-*F/NL*-*G*-*F*^ and *App*^*NL/NL*^ mice performed equally as well as WT mice. **e** and **f** During the hidden training sessions, *App*^*NL*-*G*-*F/NL*-*G*-*F*^ mice significantly spent more time and travelled a longer distance to reach the submerged platform than WT mice. **g** *App*^*NL*-*G*-*F/NL*-*G*-*F*^ mice significantly exhibited lower path efficiency relative to WT mice. **h** Swimming speed did not differ between *App*^*NL*-*G*-*F/NL*-*G*-*F*^ and WT mice. **e**–**h** Spatial learning ability in *App*^*NL/NL*^ mice was equivalent of that in WT mice. n = 17 WT (B6J), n = 11 *App*^*NL/NL*^, n  =  16 *App*^*NL*-*G*-*F/NL*-*G*-*F*^

The Morris water maze (MWM) is one of the most commonly used paradigms for assessing hippocampal-dependent spatial learning and memory in mouse models of AD [5, 15]. In this task, mice are required to use extra-maze cues to learn the location of a platform submerged below the water surface. During the initial training session, when the platform can be seen (“visible training”), the motor and visual capacities of the mice were assessed (Fig. 1a). Subsequently, the mice were trained to acquire the spatial location of a platform when the platform was not visible (“hidden training”) (Fig. 1a). One day after the sixth session of hidden training, a probe test (Probe test 1) was conducted without a platform to determine whether mice had learned the location of the platform using extra-maze cues (Fig. 1a). These mice were also subjected to a second probe test (Probe test 2) 7 days after the seventh session of hidden training (Fig. 1a).

To assess motor and visual capabilities, mice were compared across 4-day visible training sessions, during which the platform was indicated by a black cubic landmark (Fig. 1a). *App*^*NL*-*G*-*F/NL*-*G*-*F*^, *App*^*NL/NL*^ and WT mice performed equally well in these sessions: latency (Fig. 1b; *F*[2, 41] = 0.12, *p* = 0.890) and distance (Fig. 1c; *F*[2, 41] = 0.07, *p* = 0.929) to reach the platform, as well as swimming speed (Fig. 1d; *F*[2, 41] = 0.59, *p* = 0.561), were similar among genotypes. As the training progressed, all genotypes reached the visible platform in the minimum amount of time via the shortest path, as reflected by significant reductions in latency (Fig. 1b; *F*[1.3, 54] = 45.75, *p* < 0.001) and distance (Fig. 1c; *F*[1.5, 60.9] = 39.80, *p* < 0.001) across the training. Swimming speed over the course of training was significantly increased in all genotypes (Fig. 1d; *F*[3, 123] = 26.73, *p* < 0.001). These results indicate that swimming and visual abilities were comparable among *App*^*NL*-*G*-*F/NL*-*G*-*F*^, *App*^*NL/NL*^, and WT mice at 24 months of age.

Following the visual training sessions, mice were trained to find a submerged platform during 7-day hidden training sessions (Fig. 1a). *App*^*NL*-*G*-*F/NL*-*G*-*F*^ mice spent more time (Fig. 1e; *F*[2, 41] = 7.88, *p* = 0.001, post hoc, WT vs. *App*^*NL*-*G*-*F/NL*-*G*-*F*^, *p* < 0.001) and travelled a longer distance (Fig. 1f; *F*[2, 41] = 15.50, *p* < 0.001, post hoc, WT vs. *App*^*NL*-*G*-*F/NL*-*G*-*F*^, *p* < 0.001) to reach the platform than WT mice across the training. Moreover, relative to WT mice, *App*^*NL*-*G*-*F/NL*-*G*-*F*^ mice exhibited lower path efficiency, as determined by the ratio of the actual distance to the ideal path that the mice could have taken to reach the platform (Fig. 1g; *F*[2, 41] = 18.66, *p* < 0.001, post hoc, WT vs. *App*^*NL*-*G*-*F/NL*-*G*-*F*^, *p* < 0.001) [16, 17], indicating that spatial accuracy was affected in these mice. Swimming speed did not differ between *App*^*NL*-*G*-*F/NL*-*G*-*F*^ and WT mice (Fig. 1h; *F*[2, 41] = 0.36, *p* = 0.700), suggesting that difference in latency was not due to a difference in the ability to swim. Our data also revealed that *App*^*NL*-*G*-*F/NL*-*G*-*F*^ mice could still learn the location of the hidden platform, as these mice exhibited decreases in latency (Fig. 1e; WT, *F*[3.6, 58] = 4.61, *p* = 0.004; *App*^*NL*-*G*-*F/NL*-*G*-*F*^, *F*[3.4, 51.3] = 2.62, *p* = 0.054) and distance (Fig. 1f; WT, *F*[3.2, 51.0] = 7.44, *p* < 0.001; *App*^*NL*-*G*-*F/NL*-*G*-*F*^, *F*[3.8, 57.4] = 2.93, *p* = 0.030) as the training progressed. Swimming speed over the course of training was significantly decreased in WT mice (Fig. 1h; WT, *F*[6, 96] = 5.45, *p* < 0.001), while it remained constant in *App*^*NL*-*G*-*F/NL*-*G*-*F*^ mice throughout the sessions (*App*^*NL*-*G*-*F/NL*-*G*-*F*^, *F*[2.9, 43.9] = 2.18, *p* = 0.106). Taken together, these results suggest that 24-month-old *App*^*NL*-*G*-*F/NL*-*G*-*F*^ mice exhibited a significant decline in the ability to learn the spatial location of a submerged platform.

**Fig. 2:**
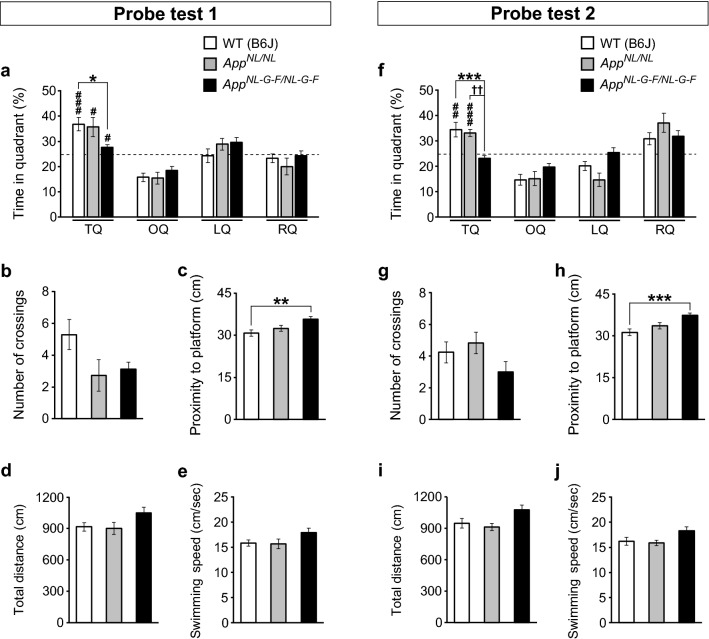
Spatial memory in *App*^*NL*-*G*-*F/NL*-*G*-*F*^ and *App*^*NL/NL*^ mice during probe tests in the Morris water maze task. **a** In Probe test 1, *App*^*NL*-*G*-*F/NL*-*G*-*F*^ mice spent less time in the target quadrant than WT mice. All genotypes spent significantly higher percentages of time in the target quadrant than predicted by chance (25%, as indicated by dotted lines). **b** The number of platform crossings did not differ among genotypes. **c** *App*^*NL*-*G*-*F/NL*-*G*-*F*^ mice exhibited significantly longer proximity to the platform than WT mice. **d** and **e** The total distance travelled and swimming speed in Probe test 1 were similar among genotypes. **f** In Probe test 2, the percentage of time spent in the target quadrant was significantly lower in *App*^*NL*-*G*-*F/NL*-*G*-*F*^ mice than in WT mice. The percentage of time spent in the target quadrant by *App*^*NL*-*G*-*F/NL*-*G*-*F*^ mice was comparable to the chance level (25%). **g** The number of platform crossings during the test did not differ among genotypes. **h** The proximity to the platform was significantly higher in *App*^*NL*-*G*-*F/NL*-*G*-*F*^ mice. **i** and **j** Total distance travelled and swimming speed were comparable among genotypes. **a**–**j** *App*^*NL/NL*^ mice had normal memory function at 1 and 7 days after the last training session. n = 17 WT (B6J), n = 11 *App*^*NL/NL*^, n = 16 *App*^*NL*-*G*-*F/NL*-*G*-*F*^. *p < 0.05, **p < 0.01, ***p < 0.001, versus WT (B6J). ^††^*p* < 0.01, versus *App*^*NL/NL*^. ^♯^*p* < 0.05, ^♯♯^*p* < 0.01, ^♯♯♯^*p* < 0.001, versus chance level

In Probe test 1 (Fig. 1a), *App*^*NL*-*G*-*F/NL*-*G*-*F*^ mice spent less time in the target quadrant than WT mice (Fig. 2a; *F*[2, 41] = 4.23, *p* = 0.021, post hoc, WT vs. *App*^*NL*-*G*-*F/NL*-*G*-*F*^, *p* = 0.025), although they still exhibited a preference for the target quadrant over non-target quadrants (Additional file 1: Fig. S1a; WT, *t*(16) = − 4.45, *p* < 0.001; *App*^*NL*-*G*-*F/NL*-*G*-*F*^, *t*(15) = − 2.14, *p* = 0.049). In addition, the percentages of time spent in the target quadrant were significantly greater than the chance level for both WT and *App*^*NL*-*G*-*F/NL*-*G*-*F*^ mice (Fig. 2a; WT, *t*(32) = 4.45, *p* < 0.001; *App*^*NL*-*G*-*F/NL*-*G*-*F*^, *t*(30) = 2.14, *p* = 0.040). During Probe test 1, although differences in the number of platform crossings between WT and *App*^*NL*-*G*-*F/NL*-*G*-*F*^ mice did not reach statistical significance (Fig. 2b; *F*[2, 41] = 2.95, *p* = 0.064), *App*^*NL*-*G*-*F/NL*-*G*-*F*^ mice exhibited significantly longer proximity to the platform than WT mice (Fig. 2c; *F*[2, 41] = 5.61, *p* = 0.007, post hoc, WT vs. *App*^*NL*-*G*-*F/NL*-*G*-*F*^, *p* = 0.005); this parameter is believed to provide a more sensitive evaluation of spatial memory performance [18, 19]. The total distance travelled (Fig. 2d; *F*[2, 41] = 2.68, *p* = 0.081) and swimming speed (Fig. 2e; *F*[2, 41] = 2.49, *p* = 0.095) in Probe test 1 did not differ between WT and *App*^*NL*-*G*-*F/NL*-*G*-*F*^ mice, suggesting that the two genotypes had similar motor capability and motivation to search for the platform. Together, these results suggest that *App*^*NL*-*G*-*F/NL*-*G*-*F*^ mice had reduced spatial memory 1 day after the last training session, but still exhibited a spatial bias toward the former location of platform.

In Probe test 2 (Fig. 1a), the percentage of time spent in the target quadrant was significantly lower in *App*^*NL*-*G*-*F/NL*-*G*-*F*^ mice than in WT mice (Fig. 2f; *F*[2, 41] = 9.03, *p* < 0.001, post hoc, WT vs. *App*^*NL*-*G*-*F/NL*-*G*-*F*^, *p* < 0.001). In addition, *App*^*NL*-*G*-*F/NL*-*G*-*F*^ mice did not exhibit a spatial bias toward the target quadrant over non-target quadrants (Additional file 1: Fig. S1b; WT, *t*(16) = − 3.30, *p* < 0.001; *App*^*NL*-*G*-*F/NL*-*G*-*F*^, *t*(15) = 1.70, *p* = 0.110). Moreover, unlike WT mice (Fig. 2f; WT, *t*(32) = 3.30, *p* = 0.002), the percentage of time spent in the target quadrant by *App*^*NL*-*G*-*F/NL*-*G*-*F*^ mice was comparable to the chance level (Fig. 2f; *App*^*NL*-*G*-*F/NL*-*G*-*F*^, *t*(30) = − 1.70, *p* = 0.099). Furthermore, although the number of platform crossings during the test did not differ between WT and *App*^*NL*-*G*-*F/NL*-*G*-*F*^ mice (Fig. 2g; *F*[2, 41] = 1.81, *p* = 0.176), the proximity to the platform was significantly higher in *App*^*NL*-*G*-*F/NL*-*G*-*F*^ mice (Fig. 2h; *F*[2, 41] = 8.94, *p* < 0.001, post hoc, WT vs. *App*^*NL*-*G*-*F/NL*-*G*-*F*^, *p* < 0.001).

Total distance travelled (Fig. 2i; *F*[2, 41] = 3.55, *p* = 0.038, post hoc, WT vs. *App*^*NL*-*G*-*F/NL*-*G*-*F*^, *p* = 0.102) and swimming speed (Fig. 2j; *F*[2, 41] = 3.13, *p* = 0.054) were comparable between *App*^*NL*-*G*-*F/NL*-*G*-*F*^ and WT mice, providing further confirmation that motor capability and motivation did not differ between genotypes. These results suggest that *App*^*NL*-*G*-*F/NL*-*G*-*F*^ mice could not retain the acquired spatial memory 7 days after the last training session.

Taken together, our findings indicate *App*^*NL*-*G*-*F/NL*-*G*-*F*^ mice exhibit definitive deficits in spatial learning and memory at the age of 24 months.

### *App*^*NL/NL*^ mice exhibit normal spatial learning and memory in the MWM task, even at 24 months of age

*App*^*NL/NL*^ mice exhibited motor and visual capabilities comparable to those of WT mice during the visible training sessions, as evidenced by the lack of a between-genotype difference in latency (Fig. 1b), distance (Fig. 1c) and swimming speed (Fig. 1d).

During the hidden training sessions, *App*^*NL/NL*^ mice performed as well as WT mice: latency (Fig. 1e; post hoc, WT vs. *App*^*NL/NL*^, *p* = 0.362) and distance (Fig. 1f; post hoc, WT vs. *App*^*NL/NL*^, *p* = 0.450) did not differ between WT and *App*^*NL/NL*^ mice. In addition, path efficiency was similar between *App*^*NL/NL*^ and WT mice across the training (Fig. 1g; post hoc, WT vs. *App*^*NL/NL*^, *p* = 0.999), indicating that both genotypes reached the hidden platform with comparable spatial accuracy. Swimming speed in *App*^*NL/NL*^ mice was also comparable to that in WT mice and significantly decreased throughout the sessions (Fig. 1h; *App*^*NL/NL*^, *F*[6, 60] = 5.40, *p* < 0.001). Taken together, these results suggest that spatial learning ability in *App*^*NL/NL*^ mice was equivalent of that in WT mice at 24 months of age.

In Probe test 1, time spent in the target quadrant did not significantly differ between WT and *App*^*NL/NL*^ mice (Fig. 2a; post hoc, WT vs. *App*^*NL/NL*^, *p* = 0.955). *App*^*NL/NL*^ spent a significantly higher percentage of time in the target quadrant than would be predicted by chance (*App*^*NL/NL*^, *t*(20) = 2.80, *p* = 0.011), and exhibited a preference toward the target quadrant over non-target quadrants (Additional file 1: Fig. S1a; *App*^*NL/NL*^, *t*(10) = − 2.80, *p* = 0.019). Neither the number of platform crossings (Fig. 2b) nor proximity to the platform (Fig. 2c; post hoc, WT vs. *App*^*NL/NL*^, *p* = 0.554) differed between WT and *App*^*NL/NL*^ mice. The total distance travelled (Fig. 2d) and swimming speed (Fig. 2e) during the test did not differ between WT and *App*^*NL/NL*^ mice. Taken together, these results suggest that spatial memory at 1 day after the last training session was equivalent in *App*^*NL/NL*^ and WT mice at 24 months of age.

In Probe test 2, the percentage of time spent in the target quadrant did not differ significantly between WT and *App*^*NL/NL*^ mice (Fig. 2f; post hoc, WT vs. *App*^*NL/NL*^, *p* = 0.907). *App*^*NL/NL*^ mice still spent a significantly higher percentage of time in the target quadrant than predicted by chance (Fig. 2f; *App*^*NL/NL*^, *t*(20) = 5.93, *p* < 0.001), and exhibited a spatial bias toward this quadrant over other quadrants (Additional file 1: Fig. S1b; *App*^*NL/NL*^, *t*(10) = − 5.94, *p* < 0.001). The number of platform crossings (Fig. 2g) and proximity to the platform (Fig. 2h; post hoc, WT vs. *App*^*NL/NL*^, *p* = 0.293) were similar between WT and *App*^*NL/NL*^ mice, as reflected by total distance travelled (Fig. 2i; post hoc, WT vs. *App*^*NL/NL*^, *p* = 0.844) and swimming speed (Fig. 2j) during the test, indicating that motor capability and motivation were comparable between these genotypes. Taken together, these results suggest that *App*^*NL/NL*^ mice had normal memory function even 7 days after the last training session. Taken together, these results suggest that *App*^*NL/NL*^ mice do not exhibit any alterations in spatial learning and memory, even at the age of 24 months.

### *App*^*NL*-*G*-*F/NL*-*G*-*F*^ mice exhibit aggressive Aβ amyloidosis and reactive gliosis, whereas *App*^*NL/NL*^ mice do not develop overt Aβ pathology in their brains

**Fig. 3:**
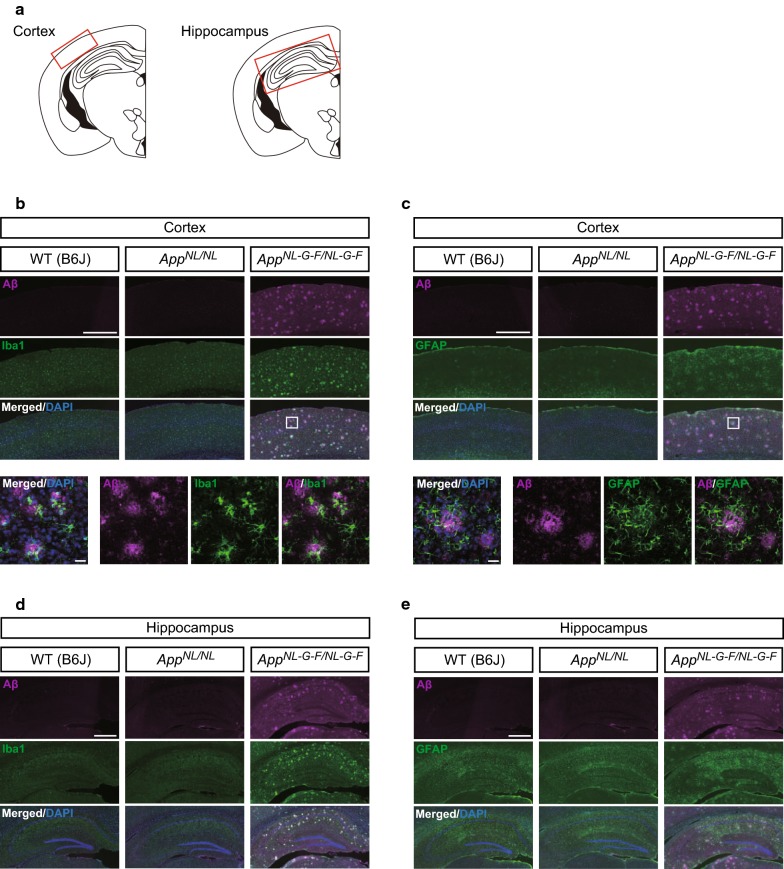
Aβ deposition and reactive gliosis in brains of 24-month-old *App*^*NL*-*G*-*F/NL*-*G*-*F*^ and *App*^*NL/NL*^ mice. **a** Red rectangles in the schematic diagrams of mouse brain slice indicated brain regions selected for capturing immunofluorescent images. **b**–**e** Representative images of cortical and hippocampal regions from coronal brain sections immunostained with anti-Aβ 82E1 (magenta in **b**–**e**), anti-Iba1 (green in **b** and **d**) and anti-GFAP (green in **c** and **e**) were shown (blue in merged images indicated DAPI staining). **b** and **c** The magnified images captured from enclosed areas by the white rectangles in *App*^*NL*-*G*-*F/NL*-*G*-*F*^ mice were shown in the bottom panels. **b**–**e** *App*^*NL*-*G*-*F/NL*-*G*-*F*^ mice exhibited extensive microgliosis and astrocytosis associated with the aggressive Aβ plaque formation in the cortex and hippocampus. **b**–**e** *App*^*NL/NL*^ mice developed neither Aβ deposition nor neuroinflammation in the brain. n = 4 per genotype. Scale bar represents 500 μm for low-magnification images **b**–**e** and 20 μm for high-magnification images (bottom panels in **b** and **c**)

To determine whether cognitive deficits are correlated with Aβ-related brain pathology, we immunostained coronal brain sections from *App*^*NL*-*G*-*F/NL*-*G*-*F*^, *App*^*NL/NL*^ and WT mice with the anti-Aβ antibody 82E1, anti-Iba1 antibody as a microglial marker, and anti-GFAP antibody as an astrocytic marker. Intense Iba1 immunoreactivity were clustered around regions of Aβ immunoreactivity both in the cortex (Fig. 3b) and hippocampus (Fig. 3d) of 24-month-old *App*^*NL*-*G*-*F/NL*-*G*-*F*^ mice, and high-magnification images in the cortex confirmed accumulation of microglia around the Aβ plaques (Fig. 3b, lower panels). *App*^*NL*-*G*-*F/NL*-*G*-*F*^ mice also exhibited intense GFAP immunoreactivity in the cortex (Fig. 3c) and hippocampus (Fig. 3e), suggestive of astrogliosis. Higher-magnification images revealed that many reactive astrocytes were present around the Aβ plaques (Fig. 3c, lower panels). Taken together, these findings indicate that *App*^*NL*-*G*-*F/NL*-*G*-*F*^ mice developed extensive microgliosis and astrocytosis associated with the aggressive Aβ plaque formation in their brains.

By sharp contrast, despite extensive analysis, we did not detect any Aβ-related pathology in the brains of *App*^*NL/NL*^ mice at 24 months of age. No Aβ deposits were detected in the cortex (Fig. 3b and c) or hippocampus (Fig. 3d and e), and Iba1 and GFAP immunoreactivities were similar between WT and *App*^*NL/NL*^ mice in both regions (Fig. 3b and c for cortex; Fig. 3d and e for hippocampus), indicating the absence of reactive gliosis in the brains of *App*^*NL/NL*^ mice. Taken together, these results suggest that *App*^*NL/NL*^ mice developed neither Aβ deposition nor neuroinflammation in the brain, even at 24 months of age.

## Discussion

In this study, we assessed the validity of *App*^*NL*-*G*-*F*^ and *App*^*NL*^ mice at an advanced age as models for investigating the early phase of AD pathogenesis, based on brain pathologies and performance in a spatial learning and memory task.

**Fig. 4:**
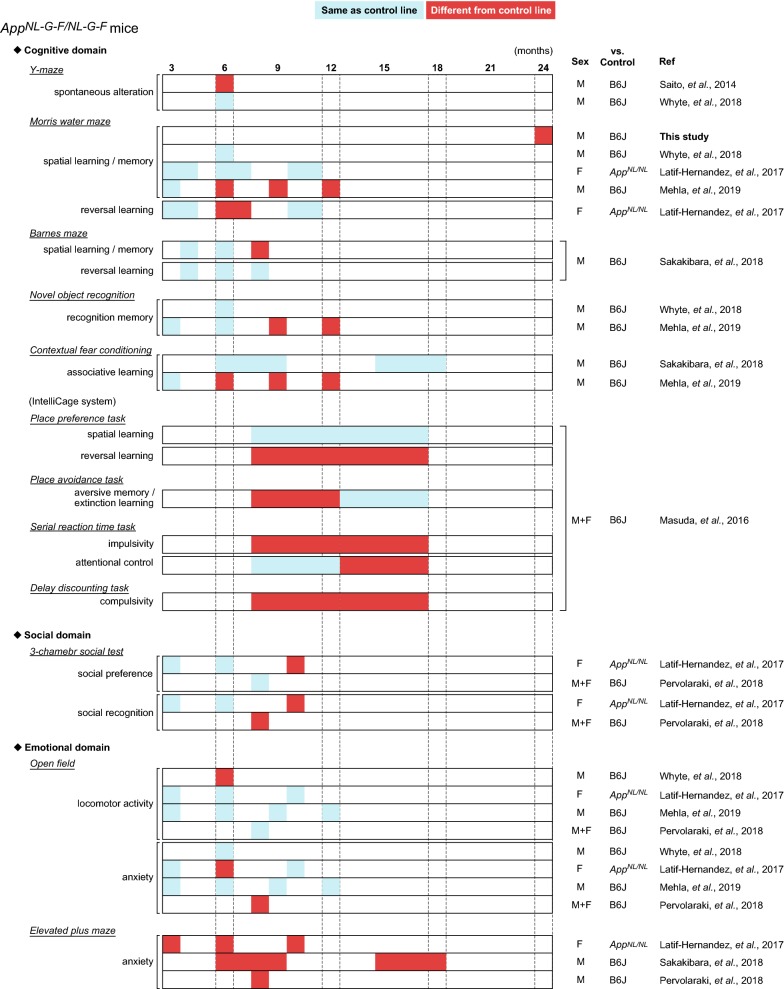
Summary of published behavioral data in *App*^*NL*-*G*-*F/NL*-*G*-*F*^ mice. Blue cells represent that *App*^*NL*-*G*-*F/NL*-*G*-*F*^ mice performed the same as control lines, while red cells represent that *App*^*NL*-*G*-*F/NL*-*G*-*F*^ mice behaved differently from control lines. Age (months) is presented in top of the figure. The “Sex” column indicates sex of mice used in the experiments; “M”, “F” and “M + F” means only male, only female and both sexes, respectively. The “vs. Control” column indicates control strain used in the experiments; B6J represents wild-type C57BL/6J strain. The “Ref” column indicates references corresponding to the data. The following references are listed in the column; Latif-Hernandez et al. [21], Masuda et al. [11], Mehla et al. [12], Pervolaraki et al. [22], Saito et al. [10], Sakakibara et al. [14], Whyte et al. [23]

**Fig. 5:**
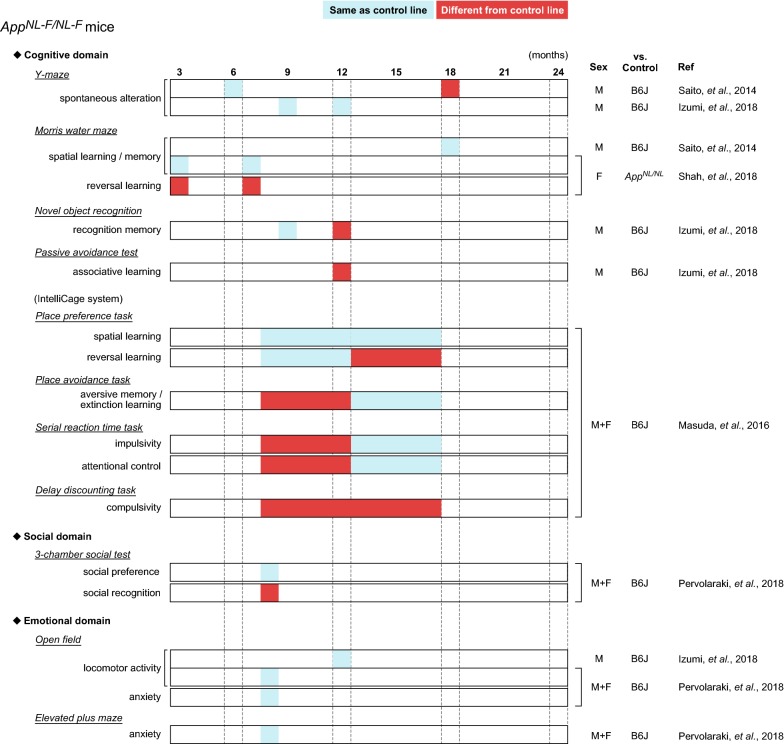
Summary of published behavioral data in *App*^*NL*-*F/NL*-*F*^ mice. Blue cells represent that *App*^*NL*-*F/NL*-*F*^ mice performed the same as control lines, while red cells represent that *App*^*NL*-*F/NL*-*F*^ mice behaved differently from control lines. Age (months) is presented in top of the figure. The “Sex” column indicates sex of mice used in the experiments; “M”, “F” and “M + F” means only male, only female and both sexes, respectively. The “vs. Control” column indicates control strain used in the experiments; B6J represents wild-type C57BL/6J strain. The “Ref” column indicates references corresponding to the data. The following references are listed in the column; Izumi et al. [36], Masuda et al. [11], Pervolaraki et al. [22], Saito et al. [10], Shah et al. [37]

**Fig. 6:**
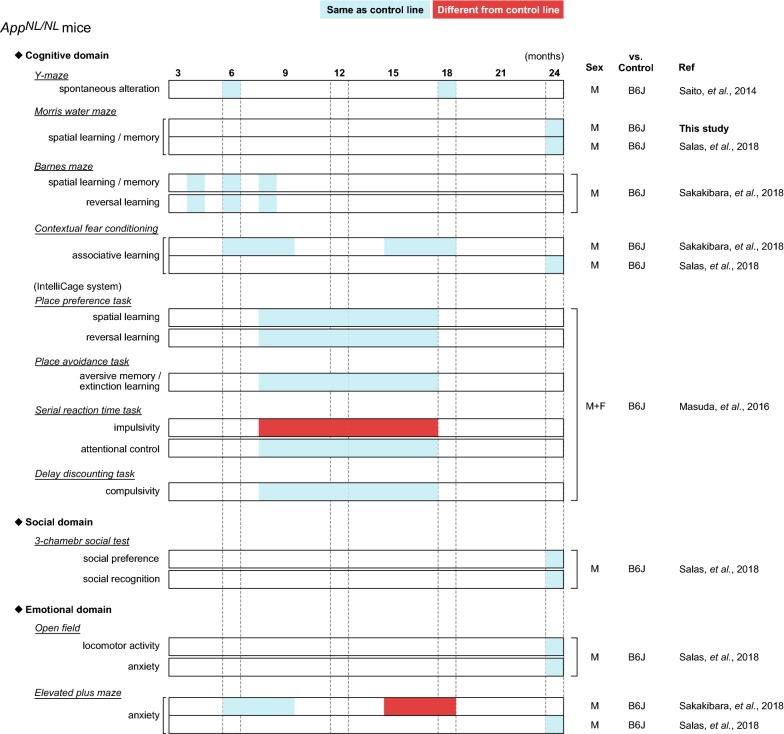
Summary of published behavioral data in *App*^*NL/NL*^ mice. Blue cells represent that *App*^*NL/NL*^ mice performed the same as control lines, while red cells represent that *App*^*NL/NL*^ mice behaved differently from control lines. Age (months) is presented in top of the figure. The “Sex” column indicates sex of mice used in the experiments; “M”, “F” and “M + F” means only male, only female and both sexes, respectively. The “vs. Control” column indicates control strain used in the experiments; B6J represents wild-type C57BL/6J strain. The “Ref” column indicates references corresponding to the data. The following references are listed in the column; Masuda et al. [11], Saito et al. [10], Sakakibara et al. [14], Salas et al. [13]

Several groups have reported behavioral alterations in cognitive and non-cognitive (social and emotional) domains in *App*-KI mice at various ages (summarized in Fig. 4 for *App*^*NL*-*G*-*F/NL*-*G*-*F*^ mice, Fig. 5 for *App*^*NL*-*F/NL*-*F*^ mice, and Fig. 6 for *App*^*NL/NL*^ mice). Among these *App*-KI mice, *App*^*NL*-*G*-*F/NL*-*G*-*F*^ mice exhibited the most severe brain pathology, including Aβ deposition and neuroinflammation (Fig. 3) [10, 11, 20]. However, the general behavioral phenotypes of *App*-KI mice described in many published studies are minimal [9], with the exception of robust alterations in emotional domains [14, 21, 22]. Accordingly, researchers have reached a consensus that the alterations in cognitive abilities of *App*-KI mice are very mild relative to the phenotypes of other overexpression models [11, 13, 21]. However, these findings are not consistent across studies: for example, in the MWM task, two groups have reported that *App*^*NL*-*G*-*F/NL*-*G*-*F*^ mice develop no obvious deficits in spatial learning and memory up to 12 months of age [21, 23], whereas another reported robust learning and memory deficits in *App*^*NL*-*G*-*F/NL*-*G*-*F*^ mice at 6 months of age [12]. In this study, to investigate whether *App*^*NL*-*G*-*F*^ mice exhibit deficits in learning and memory at an advanced age, we assessed the performance of *App*^*NL*-*G*-*F/NL*-*G*-*F*^ mice in the MWM task at the age of 24 months. Our results revealed that 24-month-old *App*^*NL*-*G*-*F/NL*-*G*-*F*^ mice exhibited a significant decline in spatial learning, and also exhibited spatial memory deficits relative to WT mice (Figs. 1 and 2), supporting the idea that sustained Aβ-related pathologies in the absence of APP overexpression are capable of inducing cognitive deficits in mice.

Another important finding from this study is that spatial learning and memory in *App*^*NL/NL*^ mice were comparable to those in WT mice at ages up to 24 months. Moreover, in sharp contrast to *App*^*NL*-*G*-*F/NL*-*G*-*F*^ mice, *App*^*NL/NL*^ mice did not develop overt brain pathologies, including Aβ deposition and reactive gliosis (Fig. 3), despite overproducing human Aβ40 and Aβ42 in their brains [10, 13]. These results indicate that introduction of the Swedish mutation in endogenous APP is not sufficient to produce brain pathology or cognitive deficits in mice, and suggest that Aβ-related pathology is required to induce these cognitive deficits. These results are consistent with recent studies reporting negligible cognitive deficits and the lack of Aβ plaque formation in *App*^*NL/NL*^ mice (Fig. 6) [10, 11, 13, 14, 24].

Familial AD mutations in *APP* and *PSEN1* genes have been utilized to develop mouse models bearing Aβ pathologies including amyloid plaques and cerebral amyloid angiopathy [2]. The first successful mouse models reproducing Aβ pathologies overexpressed human APP with either a single Swedish [25, 26] or Indiana [27] mutation. Because Aβ aggregation strictly depends on time, concentration, and Aβ42/Aβ40 ratio, dozens of mouse lines overexpressing several combinations of *APP* and *PSEN1* mutations have been created to accelerate the onset of Aβ pathologies in brains [28–30]. These mouse models have made significant contributions to AD research, however, they also suffered from potential off-target effects caused by either non-physiological overexpression of *APP/PSEN1* genes and/or combinations of familial AD mutations, which are not observed in AD patients [2, 9]. To overcome these issues, KI mouse models with familial AD mutations have been generated [10, 24, 31, 32]. To date, however, Aβ pathologies and cognitive deficits were observed only when two or three familial AD mutations were combined [33–35]. This study provides further evidence that a single Swedish mutation in *APP* is not sufficient to reproduce Aβ pathologies and cognitive deficits in mice. Our results also highlight the importance of age-associated factors to promote Aβ depositions in brains, and suggest that KI mouse models with a single familial AD mutation such as *App*^*NL/NL*^ mice may be valuable tools to truly understand the biology of *APP* mutations in AD pathogenesis. Such studies may reveal novel therapeutic targets not only for familial but also for sporadic cases of AD.

## Conclusions

*App*^*NL*-*G*-*F/NL*-*G*-*F*^ mice are an excellent model for investigating the mechanisms underlying cognitive deficits caused by Aβ-related pathologies, as well as for testing potential AD therapeutics. *App*^*NL/NL*^ mice represent a valuable model for exploring the critical factors involved in Aβ plaque formation and associated brain pathologies.

## Methods

### Animals

The original lines of *App*-KI (*App*^*NL*-*G*-*F/NL*-*G*-*F*^ and *App*^*NL/NL*^) mice on a C57BL/6J genetic background [10] were obtained from RIKEN Center for Brain Science (Wako, Japan) and maintained at the Institute for Animal Experimentation in National Center for Geriatrics and Gerontology as described previously [14]. After weaning, all mice were housed socially in same-sex groups and only male mice were used for the experiments with mixed genotypes. All handling and experimental procedures were performed in accordance with the Guidelines for the Care of Laboratory Animals of National Center for Geriatrics and Gerontology (Obu, Japan).

### Morris water maze task

All experiments were performed in a white circular pool (1.0 m in diameter; O’hara & Co., Tokyo, Japan) with a light intensity on the center of the pool of approximately 80 lx. Water temperature was maintained at 24 ± 1 °C and was made opaque using nontoxic white paint during hidden training sessions and probe tests.

First, 24-month-old mice (*n* = 11–17/genotype) were individually handled for 3 days before starting the experiment to acclimate them to the introduction and removal of the pool. After the habituation period, mice were subjected to 4-day visible training sessions (four trials per day, with an intertrial interval of approximately 15 min) (Fig. 1a), in which a platform (10 cm in diameter) was made visible by attaching a black cubic landmark. The aim of the visible training was to exclude mice with motor, visual or motivational impairments. If the mouse found the platform within a 2-min time limit, the mouse remained there for 20 s. If not, the mouse was gently guided towards the platform before staying on it. After the 20-s period on the platform, mice were placed in the cage heated by a heating pad to dry and then transported back to their home cage. The location of the platform and the start position were changed randomly in each trial. For each trial, latency to reach the platform (s), swimming distance (cm) and swimming speed (cm/s) were automatically measured using TimeMWM software (O’hara & Co., Tokyo, Japan).

Following the visible training sessions, the mice were subjected to 7-day hidden training sessions (four trials per day, with an intertrial interval of approximately 20 min) (Fig. 1a), in which the platform was placed 0.8–1.0 cm below the water surface. Four distinct objects of different geometry were used around the pool as spatial cues. At the end of the trial, either when the mouse had found the platform or when a 60-s time limit had elapsed, mice were allowed to rest on the platform for 15 s. Then, mice were placed in the cage heated by a heating pad to dry before returning to their home cage. The start position was changed randomly to avoid track memorization, while the location of the platform was fixed throughout the experiment. Latency (s) and distance travelled (cm) to reach the platform and swimming speed (cm/s) were also measured by the software. To quantify the efficiency of the strategy pursued in reaching the platform, path efficiency was calculated by dividing the distance between the first and last locations by the total distance travelled (Path efficiency = Distance between the starting point and the final point (the location of the platform)/Total distance travelled).

To confirm that this spatial task was acquired based on navigation by distal cues, two probe tests were conducted as following: the first probe test at one day after the sixth session (Probe test 1) and the second probe test at 7 days after the seventh session (Probe test 2) of the hidden training (Fig. 1a). In these tests, the platform was removed from the pool and mice were allowed to search the platform for 60 s. Time spent in each quadrant (TQ; target quadrant, OQ; opposite quadrant; RQ; right quadrant, LQ; left quadrant) (s), number of crossings over the platform location and average proximity to the platform (cm) were measured by the software. Total distance travelled (cm) and swimming speed (cm/s) were also measured to rule out the involvement of motor function and motivation to search the platform as confounding factors.

After the behavioral experiments, these mice were administered intraperitoneally with the combined agent with medetomidine (0.3 mg/kg), midazolam (4 mg/kg) and butorphanol (5 mg/kg). The whole brain tissues were collected and subjected to immunohistochemistry and other experiments.

### Immunohistochemistry

Immunohistochemical staining was performed in male mice of the 3 genotypes (*n* = 4/genotype) to examine the degree of Aβ amyloidosis and reactive gliosis in cortical and hippocampal regions (Fig. 3a). During maintenance of anesthesia with the same cocktail combination (medetomidine (0.3 mg/kg) + midazolam (4 mg/kg) + butorphanol (5 mg/kg)), mice were then perfused intracardially with ice-cold saline followed by 4% paraformaldehyde (PFA) in 0.1 M phosphate buffer (PB). The whole brains were collected and immersed in the same fixative solution at 4 °C overnight. For cryoprotection, the fixed brains were transferred into 20% and then 30% sucrose in 0.1 M PB at 4 °C until the tissues sank. Brains were sliced coronally into 25-μm free-floating sections using a cryostat (Leica CM3050; Leica Microsystems, Wetzlar, Germany). For antigen retrieval, sections were incubated with 70% formic acid at 5 min. After wash with PBS containing 0.1% Triton X-100 (PBS-T), the sections were blocked in a buffer containing 5% normal goat serum, 0.5% bovine serum albumin (BSA) and 0.3% Triton X-100 in PBS for 1 h, and then incubated overnight at 4 °C with primary antibodies in a dilution buffer containing 3% normal goat serum, 0.5% BSA and 0.3% Triton X-100 in PBS. The following primary antibodies were used: mouse anti-Aβ 82E1 (1:200; 10323; IBL, Gunma, Japan), rabbit anti-Iba1 (1:500; 019-19741; Wako, Osaka, Japan) and rabbit anti-GFAP (1:500; ROI003; SHIMA Laboratories Co., Ltd., Tokyo, Japan). After three washes with PBS-T were given, the sections were then incubated for 2–3 h with the following secondary antibodies in the dilution buffer; Alexa Fluor 594 goat anti-mouse IgG (1:500; ab150116; Abcam) and Alexa Fluor 488 goat anti-rabbit IgG (1:500; ab150077; Abcam). After three washes with PBS-T, the sections were incubated with DAPI (2 μg/ml) at 5 min and mounted in Aqua-Poly/Mount (Polysciences Inc., Warrington, USA). Immunofluorescence images of the sections were captured using a florescence microscope (BZ-9000; Keyence, Osaka, Japan) or a confocal laser-scanning microscope (LSM 780; Carl Zeiss, Oberkochen, Germany).

### Statistical analysis

As previously described [14], statistical differences between genotypes against behavioral parameters with one dependent variable were determined by repeated-measures analysis of variance (ANOVA). When necessary, Greenhouse–Geisser estimates of sphericity were used to correct for degrees of freedom. Bonferroni post hoc comparisons were used to evaluate group differences. For the comparisons of multiple means with genotypes as one independent variable, one-way ANOVA followed by the Tukey’s post hoc tests was used. One-sample *t-*test was used to compare performance on the probe test of the MWM task against chance level (25%). Differences of the percentage of time spent between target and non-target quadrants during probe tests were evaluated using paired *t*-test. Data are presented as mean ± SEM. All alpha levels were set at 0.05.

## Additional file

**Additional file 1: Figure S1**. Quadrant preference shown by each genotype during probe tests in the Morris water maze task. (**a**) In Probe test 1, both *App*^*NL-G-F/NL-G-F*^ and *App*^*NL/NL*^ mice exhibited a preference for the target quadrant over non-target quadrants. (**b**) In Probe test 2, *App*^*NL-G-F/NL-G-F*^ mice did not exhibit a spatial bias toward the target quadrant over non-target quadrants, while *App*^*NL/NL*^ mice exhibited a preference toward the target quadrant. Dotted lines indicate chance level (25%). n = 17 WT (B6J), n = 11 *App*^*NL/NL*^, n = 16 *App*^*NL-G-F/NL-G-F*^. ^§^*p*<0.05, ^§§^*p*<0.01, ^§§§^*p*<0.001, target quadrant versus average of non-target quadrants.

## Abbreviations

AD: Alzheimer’s disease
APP: amyloid precursor protein
Aβ: amyloid-β
ANOVA: analysis of variance
GFAP: glial fibrillary acidic protein
Iba1: ionized calcium binding adaptor molecule 1
KI: knock-in
MWM: Morris water maze
WT: wild-type

## References

[1] Gotz J, Bodea LG, Goedert M (2018). Rodent models for Alzheimer disease. Nat Rev Neurosci.

[2] Jankowsky JL, Zheng H (2017). Practical considerations for choosing a mouse model of Alzheimer’s disease. Mol Neurodegener.

[3] Kitazawa M, Medeiros R, Laferla FM (2012). Transgenic mouse models of Alzheimer disease: developing a better model as a tool for therapeutic interventions. Curr Pharm Des.

[4] Lalonde R, Fukuchi K, Strazielle C (2012). APP transgenic mice for modelling behavioural and psychological symptoms of dementia (BPSD). Neurosci Biobehav Rev.

[5] Webster SJ, Bachstetter AD, Nelson PT, Schmitt FA, Van Eldik LJ (2014). Using mice to model Alzheimer’s dementia: an overview of the clinical disease and the preclinical behavioral changes in 10 mouse models. Front Genet.

[6] Balducci C, Forloni G (2011). APP transgenic mice: their use and limitations. Neuromolecular Med.

[7] Gidyk DC, Deibel SH, Hong NS, McDonald RJ (2015). Barriers to developing a valid rodent model of Alzheimer’s disease: from behavioral analysis to etiological mechanisms. Front Neurosci.

[8] Rice HC, de Malmazet D, Schreurs A, Frere S, Van Molle I, Volkov AN, Creemers E, Vertkin I, Nys J, Ranaivoson FM (2019). Secreted amyloid-beta precursor protein functions as a GABABR1a ligand to modulate synaptic transmission. Science.

[9] Sasaguri H, Nilsson P, Hashimoto S, Nagata K, Saito T, De Strooper B, Hardy J, Vassar R, Winblad B, Saido TC (2017). APP mouse models for Alzheimer’s disease preclinical studies. EMBO J.

[10] Saito T, Matsuba Y, Mihira N, Takano J, Nilsson P, Itohara S, Iwata N, Saido TC (2014). Single App knock-in mouse models of Alzheimer’s disease. Nat Neurosci.

[11] Masuda A, Kobayashi Y, Kogo N, Saito T, Saido TC, Itohara S (2016). Cognitive deficits in single App knock-in mouse models. Neurobiol Learn Mem.

[12] Mehla J, Lacoursiere SG, Lapointe V, McNaughton BL, Sutherland RJ, McDonald RJ, Mohajerani MH (2019). Age-dependent behavioral and biochemical characterization of single APP knock-in mouse (APP(NL-G-F/NL-G-F)) model of Alzheimer’s disease. Neurobiol Aging.

[13] Salas IH, Callaerts-Vegh Z, D’Hooge R, Saido TC, Dotti CG, De Strooper B (2018). Increased insoluble amyloid-beta induces negligible cognitive deficits in old AppNL/NL Knock-In Mice. J Alzheimers Dis.

[14] Sakakibara Y, Sekiya M, Saito T, Saido TC, Iijima KM (2018). Cognitive and emotional alterations in App knock-in mouse models of Abeta amyloidosis. BMC Neurosci.

[15] Puzzo D, Gulisano W, Palmeri A, Arancio O (2015). Rodent models for Alzheimer’s disease drug discovery. Expert Opin Drug Discov.

[16] Illouz T, Madar R, Louzoun Y, Griffioen KJ, Okun E (2016). Unraveling cognitive traits using the Morris water maze unbiased strategy classification (MUST-C) algorithm. Brain Behav Immun.

[17] Timic T, Joksimovic S, Milic M, Divljakovic J, Batinic B, Savic MM (2013). Midazolam impairs acquisition and retrieval, but not consolidation of reference memory in the Morris water maze. Behav Brain Res.

[18] Gallagher M, Burwell R, Burchinal M (1993). Severity of spatial learning impairment in aging: development of a learning index for performance in the Morris water maze. Behav Neurosci.

[19] Maei HR, Zaslavsky K, Teixeira CM, Frankland PW (2009). What is the most sensitive measure of water maze probe test performance?. Front Integr Neurosci.

[20] Saito T, Saido TC (2018). Neuroinflammation in mouse models of Alzheimer’s disease. Clin Exp Neuroimmunol.

[21] Latif-Hernandez A, Shah D, Craessaerts K, Saido T, Saito T, De Strooper B, Van der Linden A, D’Hooge R (2017). Subtle behavioral changes and increased prefrontal-hippocampal network synchronicity in APP(NL-G-F) mice before prominent plaque deposition. Behav Brain Res..

[22] Pervolaraki E, Hall SP, Foresteire D, Saito T, Saido TC, Whittington MA, Lever C, Dachtler J. Insoluble Abeta overexpression in an App knock-in mouse model alters microstructure and gamma oscillations in the prefrontal cortex, and social and anxiety-related behaviours. bioRxiv. 2018. 10.1242/dmm.040550 PMC6765200 31439589

[23] Whyte LS, Hemsley KM, Lau AA, Hassiotis S, Saito T, Saido TC, Hopwood JJ, Sargeant TJ (2018). Reduction in open field activity in the absence of memory deficits in the App(NL-G-F) knock-in mouse model of Alzheimer’s disease. Behav Brain Res.

[24] Reaume AG, Howland DS, Trusko SP, Savage MJ, Lang DM, Greenberg BD, Siman R, Scott RW (1996). Enhanced amyloidogenic processing of the beta-amyloid precursor protein in gene-targeted mice bearing the Swedish familial Alzheimer’s disease mutations and a “humanized” Abeta sequence. J Biol Chem.

[25] Hsiao K, Chapman P, Nilsen S, Eckman C, Harigaya Y, Younkin S, Yang F, Cole G (1996). Correlative memory deficits, Abeta elevation, and amyloid plaques in transgenic mice. Science.

[26] Sturchler-Pierrat C, Abramowski D, Duke M, Wiederhold KH, Mistl C, Rothacher S, Ledermann B, Burki K, Frey P, Paganetti PA (1997). Two amyloid precursor protein transgenic mouse models with Alzheimer disease-like pathology. Proc Natl Acad Sci USA.

[27] Games D, Adams D, Alessandrini R, Barbour R, Berthelette P, Blackwell C, Carr T, Clemens J, Donaldson T, Gillespie F (1995). Alzheimer-type neuropathology in transgenic mice overexpressing V717F beta-amyloid precursor protein. Nature.

[28] Jankowsky JL, Fadale DJ, Anderson J, Xu GM, Gonzales V, Jenkins NA, Copeland NG, Lee MK, Younkin LH, Wagner SL (2004). Mutant presenilins specifically elevate the levels of the 42 residue beta-amyloid peptide in vivo: evidence for augmentation of a 42-specific gamma secretase. Hum Mol Genet.

[29] Oakley H, Cole SL, Logan S, Maus E, Shao P, Craft J, Guillozet-Bongaarts A, Ohno M, Disterhoft J, Van Eldik L (2006). Intraneuronal beta-amyloid aggregates, neurodegeneration, and neuron loss in transgenic mice with five familial Alzheimer’s disease mutations: potential factors in amyloid plaque formation. J Neurosci.

[30] Radde R, Bolmont T, Kaeser SA, Coomaraswamy J, Lindau D, Stoltze L, Calhoun ME, Jaggi F, Wolburg H, Gengler S (2006). Abeta42-driven cerebral amyloidosis in transgenic mice reveals early and robust pathology. EMBO Rep.

[31] Guo Q, Fu W, Sopher BL, Miller MW, Ware CB, Martin GM, Mattson MP (1999). Increased vulnerability of hippocampal neurons to excitotoxic necrosis in presenilin-1 mutant knock-in mice. Nat Med.

[32] Siman R, Reaume AG, Savage MJ, Trusko S, Lin YG, Scott RW, Flood DG (2000). Presenilin-1 P264L knock-in mutation: differential effects on abeta production, amyloid deposition, and neuronal vulnerability. J Neurosci.

[33] Flood DG, Reaume AG, Dorfman KS, Lin YG, Lang DM, Trusko SP, Savage MJ, Annaert WG, De Strooper B, Siman R (2002). FAD mutant PS-1 gene-targeted mice: increased A beta 42 and A beta deposition without APP overproduction. Neurobiol Aging.

[34] Kohler C, Ebert U, Baumann K, Schroder H (2005). Alzheimer’s disease-like neuropathology of gene-targeted APP-SLxPS1mut mice expressing the amyloid precursor protein at endogenous levels. Neurobiol Dis.

[35] Li H, Guo Q, Inoue T, Polito VA, Tabuchi K, Hammer RE, Pautler RG, Taffet GE, Zheng H (2014). Vascular and parenchymal amyloid pathology in an Alzheimer disease knock-in mouse model: interplay with cerebral blood flow. Mol Neurodegener.

[36] Izumi H, Shinoda Y, Saito T, Saido TC, Sato K, Yabuki Y, Matsumoto Y, Kanemitsu Y, Tomioka Y, Abolhassani N (2018). The disease-modifying drug candidate, SAK3 improves cognitive impairment and inhibits amyloid beta deposition in app knock-in mice. Neuroscience.

[37] Shah D, Latif-Hernandez A, De Strooper B, Saito T, Saido T, Verhoye M, D’Hooge R, Van der Linden A (2018). Spatial reversal learning defect coincides with hypersynchronous telencephalic BOLD functional connectivity in APP(NL-F/NL-F) knock-in mice. Sci Rep.

