# Supplementary figures and images for: Amyloid-β plaque formation and reactive gliosis are required for induction of cognitive deficits in App knock-in mouse models of Alzheimer’s disease
Source: BMC Neurosci. 2019 Mar 20;20:13. doi: 10.1186/s12868-019-0496-6 (PMC6425634; doi:10.1186/s12868-019-0496-6)

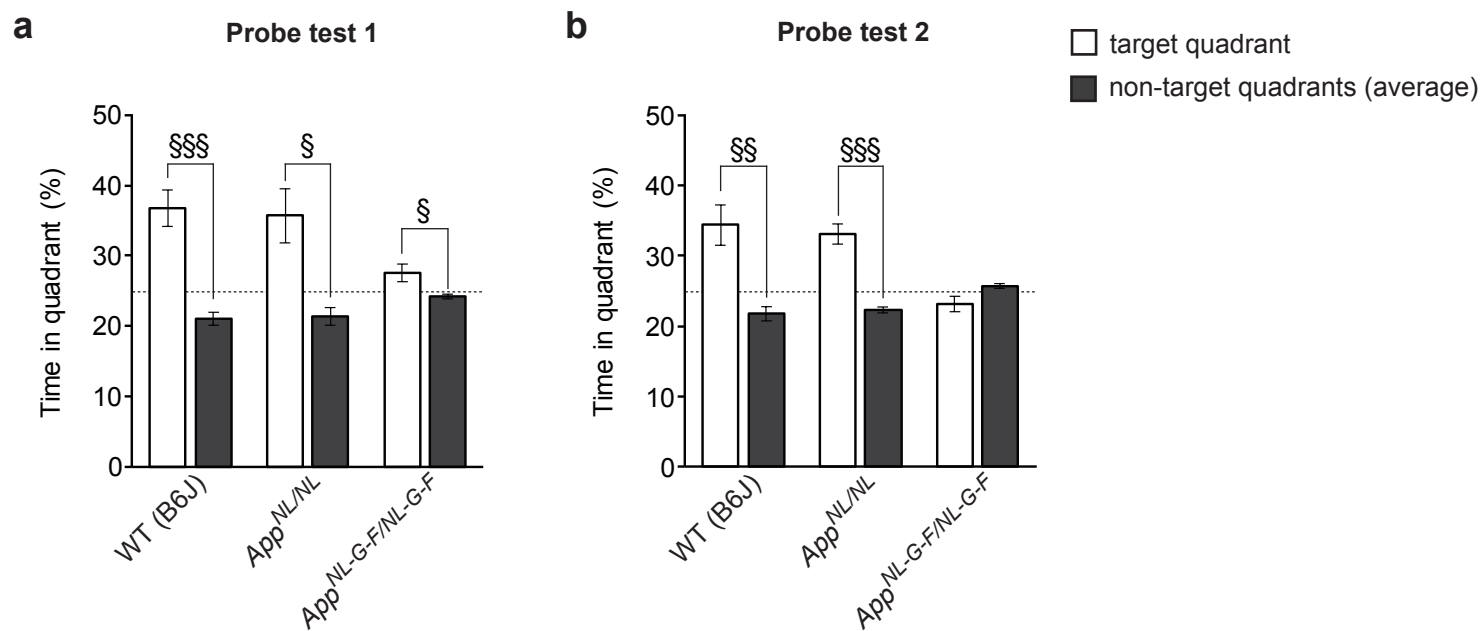

Fig. S1

Supplement: Supplementary file 1 — Additional file 1: Figure S1. Quadrant preference shown by each genotype during probe tests in the Morris water maze task. (a) In Probe test 1, both AppNL-G-F/NL-G-F and AppNL/NL mice exhibited a preference for the target quadrant over non-target quadrants. (b) In Probe test 2, AppNL-G-F/NL-G-F mice did not exhibit a spatial bias toward the target quadrant over non-target quadrants, while AppNL/NL mice exhibited a preference toward the target quadrant. Dotted lines indicate chance level (25%). n = 17 WT (B6J), n = 11 AppNL/NL, n = 16 AppNL-G-F/NL-G-F. §p<0.05, §§p<0.01, §§§p<0.001, target quadrant versus average of non-target quadrants. [file 12868_2019_496_MOESM1_ESM.pdf]
